# Spirit of the British and American Periodicals

**Published:** 1840

**Authors:** 


### Spirit of the British and American Periodicals, &c.

---

ON THE USE OF NITRATE OF SILVER IN SOME AFFECTIONS OF THE MUCOUS MEMBRANES. By ALFRED HUDSON, M.B. Physician to the Nassau Fever Hospital.*

Dr. Hudson observes that, since the Senior Editor of this Journal directed the attention of the profession to the effects of small doses of the nitrate of silver, in cases of morbid sensibility of the stomach, the remedy must have been a good deal employed. He thus refers to some who have contributed observations on the subject.

"Dr. Osborne assigns it an useful adjuvant action, as an astringent in gastralgia, with sour vomiting. Mr. Langston Parker classes it with morphia and bismuth as a *sedative* in ulceration of the stomach. Dr. Bigger gives us the testimony of Dr. Steinitz, to its efficacy in nervous debility of the stomach; and of Dr. Schneider, in dyspeptic palpitation: and a case of its successful exhibition, in this latter affection, is given by Dr. Copland, in his article "Indigestion;" and recently M. Boudin has published his observations on its effects in gastro-enterite, when given by the mouth and by enema."

1. Dr. Hudson has used the nitrate a good deal in the stomach complaints so prevalent amongst the Irish peasantry. He relates three cases of severe gastralgia in which the nitrate proved eminently serviceable. We may quote the second as a sample.

*Case.*—Michael Monaghan, aged 15, admitted February 3, 1839.—Has suffered for six months acute pain, with tenderness on pressure in the epigastric region; great distention of stomach after eating; thirst, costiveness, and vomiting of sour fluid. Pain usually commences about an hour after dinner, and continues through most of the night, preventing sleep; it is generally accompanied by vomiting of sour fluid, without food. He has been under medical treatment, but, as he says, without benefit.

Nitrat. Argenti gr. $\frac{1}{4}$.

Opii. gr. $\frac{1}{4}$.

Pulv. Rhei;

Ext. Humuli, āā. gr. i.

Ft. Pil. ter die sumenda.

Bread and milk for diet.

His farther history is, that during his stay in hospital he had but one return of the vomiting. The pain and tenderness subsided in the course of a week. The pills were discontinued on the tenth day, and on the twenty-eighth he was discharged free from complaint.

2. Dr. Hudson has resorted to it in dyspepsia, combined with sympathetic affections of other organs. He has several times given it where the painful feelings were referred to the *head,* as giddiness, especially on motion; confusion of vision; ringing noise in the ears; with, at the same time, a pale countenance and feeble circulation.

3. He has also used it in cases of nervous debility of the stomach, the primary complaint having been uterine leucorrhœa. And indeed he has seen

---

* Dublin Journal, May, 1840.

it of great advantage to the leucorrhœa itself. He details three cases; we select one.

*Case.*—Mrs. R————, aged 36, the mother of four children, was confined more than a month ago, and since she commenced moving about has been tormented by a viscid, transparent, colourless discharge, which goes off at night and returns during the day, in great quantity. She has dull pain in the loins, and gnawing at the pit of the stomach. She has had several abortions, and suffered much from the same complaint after each of them.

April 5th. She commenced taking the nitrate of silver in doses of a third of a grain, with powdered ginger and extract of hop, three times a day.

On the 15th of April she had taken ten grains of the medicine, and was quite well. She said she never got well so quickly in her life: she soon after became again pregnant.

4. Dr. Hudson has seen it very useful, as he has seen it fail, in dysentery and diarrhœa. It appeared to anwser in a case of hæmaturia. In two cases of catarrh of the bladder, and in one of hæmorrhage from the urethra it seems to have been inefficient.

He gives the following summary of its effects.

1st. A topical action upon the inflamed, congested, and ulcerated portions of the alimentary canal, similar to that which it exercises upon similar affections on the surface of the body.

2nd. A power of acting as a stimulant on the capillary circulation of different parts of the body, as of the brain and uterus.

3rd. A tonic power of the very first order.

The profession will do well to keep its eyes open to the utility of this medicine in many cases. We have for some time been in the habit of employing it, and often with advantage.

---

A Case of Hydrophthalmia successfully treated by Mercury. By James O'Beirne, M.D. one of the Surgeons of the Richmond Surgical Hospital, Dublin.

*Case.*—Mary Anne Redmond, aged 40, much exposed to weather, was admitted Sept. 24, 1839, with considerable enlargement and protrusion of the globe of the right eye, and total loss of vision of that organ.

Examined in profile, it is completely uncovered by the upper eyelid, and projects considerably more than the sound one; it is equally distended in its entire circumference, and its sclerotic portion has a bluish cast, and appears as if thinned; the relative distance between the anterior and posterior chambers is preserved. The cornea is not altered in figure, and in size bears a just proportion to that of the rest of the ball. The iris is of its natural colour, and does not project more than usual into the anterior chamber. The pupil is greatly dilated, quite motionless under the stimulus of light, and somewhat irregular at its upper margin. There is slight conjunctivitis, but a greater degree of it at the inner canthus. The lens and all the humours are perfectly transparent, but the bottom of the eye, contrasted with that of the sound one, bears a dark, blackish appearance. The eyelids are neither œdematous nor inflamed, and leave the eye perfectly uncovered. The motions of the eye-ball are not perfectly under the control of the will, and are performed with some degree of pain and difficulty.

She had contracted a severe cold on the night of the preceding 6th of January; and she stated that the affection of the eye commenced soon after with severe pain in the right eye-ball, and right supra-orbital region, followed by vivid redness of the organ; that this pain became excruciating at night; that, after continuing for several days, she lost it suddenly, and, about the same time, had the distinct sense of moderate enlargement and distention of the globe; and that the enlargement proceeded slowly, and loss of vision gradually increased, until an hour before her admission into hospital, when she suddenly observed that she was, to use her own words, " completely dark of the bad eye."

She was ordered to take a pill, containing three grains of calomel, and half a grain of opium, three times in the day: to have five leeches applied to the upper eyelid, the same number to the lower, and to be placed on low diet. Subsequently a blister was applied to the nucha. The mouth was kept affected till the 11th or 12th of October. On the 15th, we find a great improvement. A small blister was applied over the right supercilium, and an ounce of infusion of valerian given thrice daily.

On the 10th of November, vision was perfect, and the eye of the natural size and appearance. She was attacked, however, with some inflammation of the synovial membrane of the elbow, and afterwards of the knee-joint. A repetition of the calomel and opium set all right, and she was discharged cured on the 14th of December.

From a number of interesting observations on the case, by Dr. O'Beirne, we extract the following referring to the treatment.

" Mr. S. Cooper says: ' Beer has known great benefit sometimes produced by the submuriate of mercury, combined with digitalis, and a drink containing supertartrate of potassa, and borax.' In this passage we see that Beer used mercury in this disease as a sialogogue, and in no other way; and that he speaks of its use, in that form, merely as greatly benefiting, not as curing the disease. Another passage from the same work runs thus: ' At the first appearance of dropsy of the eye, many surgeons recommend mercurials, and cicuta; astringent collyria; a seton in the nape of the neck, and compression of the eye, However, Scarpa has never yet met with a single well-detailed history of a dropsy of the eye cured by these means.' Upon this passage I have only to observe, that, such as my researches on the subject have been, they support Scarpa's assertion so strongly, that I feel no hesitation whatever in asserting, that the present is the only authentic instance in which the disease has been completely cured by mercury, paracentesis, or any other means, single or combined. Indeed, Jourdan, in his article on this disease in the Dictionnaire des Sciences Médicales, after mentioning paracentesis, emetics, diuretics, and mercurial preparations, says, ' Mais il serait difficile de trouver une seule observation digne de foi, constatant l'efficacité de ces divers moyens.' Such being the actual state of the facts, it is manifest that the unexampled success of mercury in this case is owing to its having been employed, not as a diuretic, but as a sialagogue, and so as to bring the whole system, and consequently the eyeball, strongly under its influence."

---

### Some Hints on the Operative Surgery of Tumors. By Alexander Stevens, M.D. Surgeon to the New York Hospital.*

The following practical remarks are of that simple but useful character which is always agreeable to the Profession. They are plain hints given by an experienced man, the every-day knowledge applicable to every-day things, and, perhaps, more serviceable than very flashy information.

We agree with Dr. Stevens, that, after all, the most important facts to be

---

* New York Journal of Medicine, Jan. 1840.

learnt in respect to tumors, is how to remove them best by the knife. The more that is published on their external differential forms and features, and indeed on their intimate structure, the more rather than the less obscurity is thrown around them; and the multiplication of species and varieties has really seemed to be a multiplication of difficulties. It is to the operations on tumors, therefore, that Dr. Stevens turns, and we direct, for a few minutes, our readers' attention. We shall extract such hints as appear to claim our notice.

*Extirpation of Encysted Tumors of the Scalp or Eyelids.*

"The most common mode of extirpating these tumours is by dissecting them out; but this is not always easily done, especially if the tumour be very small. I have known half an hour occupied in removing a tumour, not larger than a pea, from the upper eyelid. Sir Astley Cooper advises that they should be cut into, and then torn out. If the first of these operations is attempted, the surgeon should be quite sure that he does not begin to dissect around the tumour until he has laid it quite bare. But I prefer the other method, and this is the way of proceeding that I would recommend:—At the first incision, I would cut freely into the sac of the tumour, seize the sac with the forceps, and pull it away either at once, or in different portions. If the sac resists, it will be because you have seized with the forceps one or more of the layers of cellular tissue which are always found surrounding the sac, and which are occasionally dense and strong. The connection of the sac with these layers is loose, but they adhere closely to each other. A few months since I removed, in two or three minutes, six of these tumours from the head of a young gentleman of this city. The rule, therefore, is this:—*cut into the sac and turn it out;* but do not attempt to tear away any thing else with the sac.

If it should happen that any portion of the sac has formed strong adhesions to the surrounding parts, an occurrence which is extremely rare, it is proper that you should understand that a perfect cure may be obtained by destroying the internal membrane (which is seldom thicker than parchment,) with a slight application of the kali purum, or of the nitrate of silver."

*Extirpation of Solid Tumors.*

Dr. Stevens remarks that, in the removal of these, unless they be malignant, and the abstraction of much of the circumjacent parts is necessary, there is one rule which should be written in letters of gold. "Did I say one rule? Let me rather say two rules, the first of which is this:—*cut down to the tumour.* This may seem to be a simple matter, so simple that the necessity of it must occur to every one. Be this, however, as it may, I do aver that in some hundreds of tumours which I have seen operated upon, and often by very skilful surgeons, the tumour has seldom been fairly exposed and laid bare before its dissection has been commenced. Vessels have been unnecessarily divided, and the whole operation has been protracted by the loss of blood, and the necessary application of ligatures to the arteries. How this happens I will now attempt to explain.

Let us take for illustration, the very common case of an enlarged lymphatic gland, in the neck. In its normal condition, this gland is supplied by one principal nutritive artery, and it is surrounded by an indefinite number of layers of cellular tissue. The layer next the gland embraces it like a shut sac; the exterior layers in contact with this, diverge and surround the adjacent parts. When the gland becomes enlarged from hypertrophy, or by becoming the seat of malignant deposits, the innermost layer of cellular tissue forms a sac, and its connection with the gland is usually loose, so that it may be readily stretched, or torn with the finger or the handle of the scalpel. The outer layers are also, in general, loose, and capable of being torn in the same way; but the manner in which they are applied to the gland, or rather to its sac, is worthy of particular attention, as affording a clue to the difficulties which are often encountered in these operations. The external layers of cellular tissue which cover the gland become, in the progress of its enlargement, stretched upon the exterior surface of the sac, being sometimes adherent to it, and to one another; from this point they diverge, passing to the anterior surface of some muscle, nerve, or blood-vessel, or to the posterior surface of some of these or of other organs. The tumour itself, in the meanwhile, receives no new vessel, other than that which originally supplied it, even though it may have grown so as to completely surround the carotid artery, the internal jugular vein, and their branches. Even in this case, the proper sac will be found interposed between these parts and the tumour. These vessels are, in other words, pressed into the side of the tumour, which, with its sac, becomes folded around them;—thus, strictly speaking, they form no part of the tumour, being exterior to the sac.

Keeping in mind the close application of several layers of cellular tissue, over the most superficial portion of the tumour, (the first and greatest enlargement of the tumour being in this direction, because it is there least opposed in its progress by the pressure of the surrounding parts,) and the separation of these layers on the lateral and deep-seated portions of the tumour, it is easy to understand:—

1st. That important blood-vessels, nerves, and other organs may be brought into close proximity to the morbid growth without absolutely touching it.

2nd. That if the surgeon, in cutting down upon the tumour, does not divide each and every layer investing the tumour before he begins to dissect around it, he cuts outside the sac, gets into some of the folds of cellular tissue, and encounters parts which ought not to be meddled with. He finds his knowledge of normal anatomy of little service to him; he gets away from the tumour, and makes a tedious and bloody operation in a case, where a different method of proceeding would have made every thing plain and easy.

Finally, when the tumour is removed and examined, folds of cellular tissue, perhaps portions of muscle, or of other parts, are found to have been removed with it, which can be torn off, and that very readily, from its external surface. Had the surgeon, in the first instance, cut down to the tumour after dividing every layer investing it, no more difficulty would have been experienced in tearing these layers from the tumour before it was removed than afterwards."

Sometimes, so transparent are the layers of cellular tissue, that it is very difficult to tell when the tumor *is* exposed. It is better for a young surgeon, and even for an old one, if he has any doubt in the matter, to cut a little into the tumour, in order to be sure that he has fairly cut down to it. Having reached the tumour, Dr. S. continues, if the cellular tissue can be torn by the fingers or by the handle of the knife, tear it; in cases where it cannot be so torn, cut in this manner: put the tumour upon the stretch, and cut lightly upon it near its points of attachment. Thus you avoid the possibility of any large blood-vessel or nerve being brought under the edge of your knife without being seen.

If the tumour is very large, or is deeply seated, it will, sometimes, be advisable, after having separated the attachments of the exterior portion of it as deeply as possible, to remove this portion. The removal of the remaining portion is thus much facilitated.

"In this manner, I safely removed a large tumour situated beneath the mastoid muscle, and which embraced the ninth pair of nerves in one part, and the common carotid artery, the internal jugular vein, the par vagum and œsophagus in another part; after very little cutting the sac was separated from these parts. I have never taken up the carotid artery for the removal of a tumour in the neck or face, nor do I believe that it is ever necessary. If the principles already laid down are carefully observed, there will be no danger of hemorrhage, nor yet of sloughing, from the nerves and blood-vessels being extensively laid bare;—laid bare, indeed, they are, but their sheath still covers them, and is sufficient for their nourishment. I have, on several occasions, left them plainly exposed, from the sternum to a point above the bifurcation of the carotid artery, and have never known secondary hemorrhage to follow."

"In some cases of malignant tumours, not only the superficial, but other portions of the sac will be found closely adhering to the adjacent parts. If the tumour is in the vicinity of important parts, as in the axilla or neck, the plan I would recommend is this:—cut down until the knife fairly enters the diseased parts, then, by the sight and touch, decide where the tissues, adjacent to the disease, are entirely healthy; make a slight incision into them on the distal side of the tumour; continue to separate them with the handle of the scalpel and the finger. If you are among healthy parts, as you proceed the cellular and other tissues will yield to a very moderate degree of force; the separation of the veins, arteries, and, lastly, the nerves, will require more force, increasing in the two last named. These parts will be felt like strings holding the tumour, and are not easily separated. Be careful not to use much force in the separation of a large artery, and still more in the separation of a large vein. It is a great mistake to suppose that arteries when torn never bleed: I have often seen them bleed, *per saltum*, after having been torn by the finger. Still, they do not bleed so freely as when cut, and, moreover, their orifices are usually easy to be found, and as easily secured. They also stop bleeding much sooner, if an attempt is made to check the hemorrhage by pressure. A nerve no larger than a silk thread is half as strong; yet I have broken them when nearly as large as a small crow quill. My practice is to bring the resisting cord, be it vein, artery, or nerve, into view upon the palmar side of the fore-finger of my left hand, and then to seize it with the forceps, and divide it half an inch on the distal side of that instrument. If it is an artery, its patulous mouth will be seen, and a ligature may be applied before the forceps is removed. Thus you will conform to the *second rule*, that is, to remove the whole tumour and nothing more."

*Dangers of Operations.*

Dr. Stevens enumerates as the dangers to be immediately apprehended and guarded against—hæmorrhage—the introduction of air into the veins—and exhaustion.

*Hæmorrhage.*—On venous hæmorrhage we need say nothing.

"Arterial hemorrhage may be diminished by tearing the vessels from the tumour. I have seen some surgeons tear the tumour itself out: this cannot always be done except to a limited extent, because a large number of parts are thus put upon the stretch at once. The better way is to hold the tumour, and tear off its investments, one portion at a time, with the fingers or with a strong pair of forceps: this method is also less painful than the former. Sometimes a vessel will retreat behind the ramus of the lower jaw, or into the axilla, and give rise to a troublesome bleeding. As these are usually the last attachments to be divided, it may be prudent to tie them before this division is made. I would also advise you always to divide and to secure the trunks of arteries, before dissecting among their branches. If you neglect this rule, you may cut and tie the same vessel half a dozen times, as I have often seen done. This is the reason some surgeons are constantly encountering tumours of extraordinary vascularity; this vascularity being, in fact, simply owing to their wandering away from the sac of the tumour, and dividing the vessels at each successive cut nearer and nearer to the heart."

An important means of diminishing hæmorrhage, in the removal of large tumours, is to subject them, for some hours previous to the operation, to the influence of cold applications.

*Introduction of Air into the Veins.*

"I have met with this occurrence only once in my practice, and that was in this Hospital about ten years since. I was in the act of removing the last of several of the deeper chain of lymphatic glands of the neck, which had become enlarged so as to interfere with the functions of deglutition and respiration, and was cautiously using the knife about half an inch on the outer side of the internal jugular vein. After a slight escape of venous blood, I heard a noise like that produced by drawing up with a syringe the last drop of water in a vessel. I immediately placed my finger over the spot from which the blood had issued, not being able to discover any orifice; and looking the patient in the face, asked him how he felt, he answered, 'very well.' Marks of consternation were visible around me, and many suggestions were made which I did not heed, but calling for an eyed probe, I directed a ligature to be passed through it. I applied to the internal jugular vein two ligatures,—one above, the other below the wound, directing them to be successively tightened. I then removed my finger, and proceeded with the operation. No bad consequences followed the application of the ligatures. The wounded vein appeared to be a branch of the internal jugular, but I did not think it safe to pass a ligature directly round the divided vessel, not liking to run the hazard of removing the pressure of my finger."

Dr. Stevens objects naturally to operating on a female about her menstrual period. He recommends the operator to have the instruments he wants within his *own* reach. He likes, in great operations, to have the assistance of a judicious medical practitioner, a personal friend, if possible, of the patient, to console, to watch, to support him.

"An adult, with ordinary powers of endurance, will, generally, sustain an operation of the average severity, during protracted suffering of one hour's duration,—rarely much more than this. A clammy skin, with coldness of the extremities, and a soft, thready pulse, indicate alarming exhaustion of the vital powers. But an experienced surgeon will judge most accurately from the expression of the countenance, from the eye and mouth especially :—the former partially loses its lustre, the latter becomes relaxed, until, finally, the eyes are turned upward, and the jaw falls, indicating an almost hopeless condition. The voice, also, is an index of the degree to which the vital powers are sunk; its tones become more and more feeble, until, at length, the patient can only speak in a low whisper, like one in the collapsed stage of cholera, and finally ceases to articulate at all. On the first approach of this state of things, I would advise you to give your patient a few minutes' respite. I give you the above indications, as being the only ones that occur to me as capable of being conveyed by language; your own observation will hereafter enable you to determine their real value. It is also important for you to know that a patient will endure a long operation much better by being allowed two or three short intervals in which to rally during the progress of an operation, it being more easy to prevent him from sinking, than to raise him from extreme prostration."

Finally Dr. Stevens counsels the surgeon never to undertake an operation against his own judgment, nor if possible assist at one.

---

### Dr. Law on Disease of the Brain, dependent on Disease of the Heart.*

M. Legallois first drew attention to the fact, a striking and, practically, an important one, that hypertrophy of the heart disposes to apoplexy. Legallois

---

* Dublin Journal, May, 1840.

has been followed by others, and the doctrine has for some time been firmly established. The object of Dr. Law, is to extend the proposition, and to lead us to believe that disease of the brain may not only be occasioned by too great, but also by too little an impetus or quantity of blood, the result of cardiac disease.

He lays down the following propositions, which he supports with several cases and with much ability. For the facts and arguments we refer to our esteemed contemporary. The propositions themselves are sufficiently intelligible in their naked form. They are as follows :—

1st. The pathology of the brain is in many instances intimately connected with, and dependent upon, pathology of the heart.

2nd. To limit the pathological relation existing between these two important organs to apoplexy, the result of hypertrophy of the left ventricle of the heart, is to narrow it much within its true limits.

3rd. Ramollissement of the brain occurs in connexion with diseases of the heart, whose effect is either directly or indirectly to diminish the flow of blood to the head.

4th. This cerebral lesion may be connected with either disease of the aortic or mitral valve.

5th. Hypertrophy of the left ventricle of the heart, in order to produce apoplexy, must depend upon some impediment to the circulation, placed at a greater distance from the heart than the origin of the vessels which convey the blood to the brain.

6th. When ramollissement of the brain occurs, in connexion with an imperfect or patulous condition of aortic valves, the close analogy that we trace between the physical signs and constitutional symptoms of this lesion and hæmorrhage, as well as the results of treatment, render it very improbable that the disease of the brain is the result of too much blood driven to it, and with undue force.

7th. When ramollissement of the brain occurs, in connexion with disease of the mitral valve, the state of the pulse, which, as a diagnostic mark of this lesion, is habitually small, precludes the idea that the cerebral lesion is produced under the usual conditions of inflammation.

8th. While ramollissement of the brain occurs as a result of inflammation, hyperemia, &c., it occurs also under diametrically opposite circumstances.

9th. To confound such opposite modifications of disease, and to apply to them the same treatment, must necessarily lead to the most mischievous practical results.

10th. The circumstances under which we have seen ramollissement of the brain to take place, seem to identify it with gangrene, or death of a part consequent upon a diminution of its due supply of blood.

---

### HOSPITALS OF MALTA.

There are four hospitals in Malta, one naval, one military, and two civil. One of the last is in Valetta, and the other in Citta Vecchia or Notabilé, near the centre of the island. There is a civil hospital, also, in Gozo. At Floriana, adjoining Valetta, there is an asylum for the insane, and the town last mentioned is furnished with a public dispensary. The civil hospital in Valetta is as well furnished and as neatly kept as most of the institutions of the kind in England and France.

A University was established in the island, by the Jesuits, in the year 1592. It has included a medical department ever since its commencement. The Medical Faculty is composed of five Professors. The chairs are, 1st. Anatomy and Surgery; 2d. Physiology and Pathology, including Theory and Practice; 3d.

Botány, Medical Jurisprudence, and Natural History; 4th. Obstetrics; and 5th. Chemistry and Materia Medica.

At the commencement of the year 1839, the organisation and discipline of the University were remodelled, and some changes were made in the Medical School. The medical students receive constant instruction in the hospitals.

These particulars are extracted from a paper by Pliny Earle, M.D. in the American Journal of the Medical Sciences, for February, 1840.

---

### Section of the Hamstring Tendons, for Contracted Knee-joint. By Dr. Burleigh Smart.*

Case.—A scrofulous boy nine years old. The knee of the left leg began to enlarge, on each side of the ligament of the patella, six years ago, accompanied with a slight lameness in walking,—no pain, tenderness or redness. At this time there was a perceptible tightness of the tendons in the ham, and a disposition to flexion of the limb when in a recumbent posture. This thickening of the integuments of the joint, was followed by an apparent enlargement of the articular extremities of the bones of this joint. The enlargement, however, never became very great, sufficiently so, to render it distinctly perceptible at sight. About two years since, the boy was found to be affected with an angular anterior curvature of the spinal column, at the junction of the lumbar, with the sacral vertebræ; and the knee-joint more flexed, other appearances of the joint remaining the same. At this time the general health, always good before, appeared to be affected—some loss of flesh and strength, and restless nights. By the use of moxa and caustic issues, each side of the angular projection, kept open for about eight months, and the internal use of sarsaparilla, soda, and the hydriodate of potassa, the disease of the spine was apparently cured. But now there was a considerable degree of stoop, or inclination forward, given to that portion of the trunk above the pelvis. In this state Dr. Brown, of Boston, being consulted, advised the application of a mechanical apparatus, to be constantly worn, together with the use of an inclined plane; which seemed to afford important aid in rectifying the forward inclination of the trunk. But as the straightening of the spinal column progressed, an increase of the flexion of the knee was observable.

For the period of about five years, this lad, in walking, has been able to bring only the toes and ball of the foot to the ground; the heel not coming to the ground, in the ordinary step, by the distance of between two and three inches, the latter part of that period of time. The leg has been a little smaller than the other, below the knee, ever since the commencement of the distortion.

On the 15th of November, the tendons of the biceps flexor cruris on the one side of the popliteal depression, and of the semi-tendinosus and semi-membranosus on the other, were divided, about two inches above their insertion. The operation was performed with a slightly curved and sharp-pointed bistoury, which was introduced with the flat surface parallel and close to the inner side of the tendons, with the point upwards, and penetrating sufficiently deep to hook the point of the instrument under the tendon, by gently depressing the handle and turning the blade of the bistoury on its own axis, until it revolved a quarter of a circle, and the edge was presented transversely to the tendon. Then, by a gently sawing motion, depressing the handle, and at the same time elevating the point of the instrument, the parts were divided with but very trifling pain.

The punctures were accurately closed by court and adhesive plaster, and a

---

* American Journal of Medical Sciences, Feb. 1840.

compress and bandage were applied, with a crooked splint outside the bandage; the limb having been extended about two inches, measured at the heel, before the dressings were applied. The flexion of the leg previous to the operation was about 40 degrees.

The 8th day subsequent to the operation, no pain or troublesome inflammation having supervened, a carved wooden splint, with its cavity padded, and having a hinge joint at the knee, by which the two parts were connected, was applied and confined with broad padded straps passing from one side of the splints to the other, fastening with buckles above and below the knee. On the posterior surface of the splints was fixed a screw, connecting the two splints; by the daily turns of which, the limb was gradually extended until it became very nearly straight, which was effected in a fortnight after the extending apparatus was applied, and three weeks after the operation.

This patient commenced walking as soon as the extending apparatus was applied. He is now able to walk with or without it, bearing his weight on the entire length of the foot; he can place the heel on the ground at every step, which he had not been able to do for about five years.

---

### OBSERVATIONS ON THE EMPLOYMENT OF CIMICIFUGA IN THE TREATMENT OF CHOREA.*

The *cimicifuga,* known in America under the common name of *black snake root,* has been recommended by Dr. Young of Pennsylvania, as a remedy for chorea. Dr. Kirkbride has just published seven cases in which he tried it. He concludes them by observing:—

"We feel satisfied of the value of the cimicifuga, in the treatment of chorea, and disposed to attribute to it powers in some other affections in which we have not yet had an opportunity of giving it a satisfactory trial. After the details we have given, it is hardly necessary to say, that we do not look upon it as a specific in chorea. We have scarce ever met with a case where the primary treatment was not plainly indicated by the disordered digestion, the loaded bowels, the pain or heat of the head, and the languid circulation of the skin. But it is also right to state, that where these symptoms have been properly treated, the involuntary muscular movements, have often continued unchanged, until after the employment of the black snake root. Purging we have always used before the cimicifuga, and general frictions with salt or the flesh-brush, and pustulation with croton oil over the spine, we have believed to be of much value in the chronic cases. Of the two preparations we have employed, we are disposed to give the powdered root the preference, and now regret that we did not administer large doses in that form in our fifth case, where the decoction certainly had no effect."

---

### CURE OF SQUINTING BY DIVISION OF THE RECTUS INTERNUS MUSCLE.

This operation is beginning to attract attention, indeed it may be looked on as already an established one. Proposed, and first, we believe, executed by Dieffenbach, it has been taken up by Dr. Franz, and by several surgeons in town, and the cases in which it has been resorted to are respectable in appearance and number.

As Dr. Franz's cases have had the priority of publication, we think it only fair to notice them. We shall select the first case.

---

* American Journal Medical Sciences, Feb. 1839.

Case.—Louisa M'Cleish, aged 18, affected with squinting since the age of two years. "On examination, I found the left eye slightly inverted, but the right eye so much turned inwards, that one third of the cornea was hidden by the inner canthus. She stated that the right eye was larger than the left, having been frequently told so by others; but this I found to be an error, probably arising from the circumstance that so little of the cornea was visible, whilst a large portion of the sclerotica presented itself. With considerable exertion of will she was scarcely able to direct the right eye so as to look straight before her, and could not in the slightest degree move the globe towards the exterior angle. When not under the influence of the will this eye instantly returned to its usual position inwards. On closing the left eye she could only distinguish large objects, and was not able to read even the largest print.

Having provided myself with three assistants, the patient, whose left eye was closed by a bandage, was seated facing the light with her head inclined backwards, in which position it was retained by an assistant, who at the same time fixed the upper eyelid with a retractor. A second assistant kneeling before the patient, fixed the lower lid by means of a retractor, held in his right hand: the eyelids being by these means well secured and drawn asunder, I perforated the conjunctiva at the inner corner of the eye close to the eye-ball, with a small and very sharp hook, and gave it to the second assistant to hold with his left hand, with which he was, by means of this hook, to draw the globe outwards. I next made a semicircular incision with a scalpel through the conjunctiva, about six lines in length; then dissecting through the subjacent cellular tissue, exposed the internal rectus muscle, and terminated the operation by dividing it close to the sclerotica with a very small pair of curved scissors, the one blade of which was passed beneath the muscle. The duty of my third assistant was to hold the patient's hands, reach me the instruments, and attend to the bleeding, which in this case was very inconsiderable. On the removal of the hook from the conjunctiva, the eye-ball was no longer inverted, but stood in the proper position, the pupil occupying the centre of the eye. The edges of the wound in the conjunctiva were brought together by the motions of the eye itself, which were perfectly free in all directions, except inwards. Cold water dressings were ordered, and a common draught. The patient then walked home."*

Mr. Mayo, Mr. Liston, and others, have performed this operation. It is probable that the simple division of a single muscle will be found inefficient in some, if not in many, cases, and no doubt experience will suggest modifications in the method.

---

### Studentships in Anatomy.—College of Surgeons.

It is with great gratification that we publish the following.

The President and Council have great pleasure in announcing to their members, that three Studentships in Human and Comparative Anatomy have been instituted by the College, to be held respectively for the term of three years, with the annual stipend of one hundred pounds attached to each studentship; and that, at the instance of the Director-General of the Medical Department of the Army, the Physician-General of the Royal Navy, and of the Chairman of the Honourable East India Company, the General Commanding the Army in Chief, the Lords Commissioners of the Admiralty, and the Court of Directors, have, with the view of promoting the objects of the College, been pleased to place at the disposal of the President and Council an Assistant-Surgeoncy, in each service, once in three years, for such of the said Students as may be considered worthy of these honourable distinctions.

The President and Council have also the pleasure to announce, that, with the view of

---

* Med. Gaz. April 17, 1840.

rendering the prizes granted by the College more worthy of competition amongst their younger members, they have augmented the Collegial (Triennial) Anatomical Prize from thirty to fifty guineas; and have added ten guineas to the like sum, allotted by its founder, to the Jacksonian (Annual) Surgical Prize.

(By Order) Edmund Belfour,
May, 18, 1840. Secretary.

---

Return of Cases of Hydrocele treated at the Native Hospital, Calcutta, after the Plan of J. R. Martin, Esq. by a retained injection of Solution of Tincture of Iodine, from 9th March 1832, to 31st December, 1839.

| | | | |
|---|---|---|---|
| From 9th March to the 31st December | 1832 | .. | 32 Cases. |
| For the year | 1833 | .. | 49 „ |
| „ „ | 1834 | .. | 86 „ |
| „ „ | 1835 | .. | 121 „ |
| „ „ | 1836 | .. | 332 „ |
| „ „ | 1837 | .. | 528 „ |
| „ „ | 1838 | .. | 585 „ |
| „ „ | 1839 | .. | 660 „ |
| Grand Total | | .. | 2393 Cases. |

Of the total cases treated there were :—

| | |
|---|---|
| Hindoos | 1,265 |
| Mahomedans | 1,076 |
| Christians | 52 |

The failures from first to last would appear to be about one per cent., or rather under it. There were, of reported failures, six cases, up to 1837, of which three were out of twelve cases treated experimentally by undiluted port wine instead of the iodine solution. Nine cases were treated at once successfully, up to 1837, by the iodine solution, in which the solution of port wine and sulphate of zinc had failed. Of late years it would appear that a large proportion of those treated are natives of Orissa, where hydrocele would seem to be endemic. No ordinary complication has interfered with the operation, and it has now superseded all others in India.

N.B.—The details of treatment will be found at pages 204 and 411 of the 7th volume of the Medical Transactions of Calcutta, and at page 12 of the Quarterly Journal of the Medical and Physical Society of this City.

P. O'BRIEN, 1st. Assist. Native Hospital.
(A true copy) J. R. MARTICE.
*Native Hospital Calcutta, 1st. January* 1840.

---

Unfermented Bread.

The profession is aware that Dr. Whiting, a few years ago, took out a patent for bread prepared without yeast, by combining muriatic acid and carbonate of soda, so as to produce common salt in the dough. From some cause or other, Dr. Whiting's bread did not take with the public, though it certainly was a wholesome composition for weak stomachs. Mr. Dodson, of Blackman street, Southwark, has much improved upon Dr. Whiting's formula. We have been using his bread, prepared in the same manner as Dr. W's., but with larger proportions of the muriatic acid and soda, for some months, as have several of our friends and patients. We can speak to its wholesome qualities, and to the ease with which it is digested, without turning acid on the stomach. We recommend our dyspeptic friends to try it—especially the brown bread, of which all our patients highly approve. Mr. D. has extended the same process to several species of pastry, and,we think, with much success.

---

### Testimonial to Sir B. C. Brodie, Bart.

All who are anxious to see professional honour and talent appreciated, will be glad to find that a subscription has been set on foot, for the purpose of presenting a Testimonial to Sir Benjamin Brodie, on his retirement from the office of Surgeon to St. George's Hospital. The motives which led to that step were so conscientious, and the step itself so unusual a sacrifice of emolument and influence to a fine sense of propriety, that it would be a wrong to Sir Benjamin Brodie individually, and a more serious wrong to the best interests of the profession, were it allowed to pass unsignalized by some exhibition of public feeling. Sir Benjamin Brodie's retirement from the hospital is, unfortunately, the termination of the more active and onerous part of his career, and in receiving a testimonial on such an occasion, he may be said to obtain during his life-time something like posthumous fame. And who can merit that more than Sir Benjamin Brodie has done? With a zeal that never flagged, and industry that would not submit to the restrictions demanded by a constitution far from robust —with a singleness of purpose, we may add, of heart, which those who know him best, are the foremost to acknowledge—with a quickness of observation, precision of ideas, and correctness of conclusion that are admirably qualified for scientific investigations, Sir Benjamin Brodie commenced and carried on, with a success that needs no chronicle, those pathological researches which have been the foundation of his fame and fortune. A Testimonial to the man, is affixing the seal of public approbation on the qualities that made him what he is, qualities that, so esteemed, will become the heritage of younger minds. And what can be more agreeable than rewarding past and fostering future merit in the same breath?

On the students, young and old, of St. George's Hospital, Sir Benjamin Brodie has a peculiar claim. Who does not know the urbanity of his demeanour, the punctuality of his attendance, the spirit of observation that he promoted, the studies that he encouraged, the example that he set? No man was, in every sense, more thoroughly the student's friend than Sir Benjamin Brodie has been. On the look out for merit, he always encouraged, always fostered, and not seldom *fed* it. Nor was his the cheap encouragement of words only, the open purse has often seconded the earnest exhortation; and the aid which he has given to the professional struggle of the young man, has been exceeded only by the munificence which relieved the distresses of the old.

To those who *know* Sir Benjamin Brodie, this picture will not seem overcharged. If any think it so, it is because a modesty, which in such cases amounts to a fault, and an aversion to publicity which is unjust to its possessor, have but too effectually concealed from common observation the most liberal feelings and most generous acts.

We trust that the subscription list will evince the respect that the profession entertains for one who still adorns it. May this Testimonial, the crowning honour of one scene of usefulness be the introduction to another. May the ease and dignity of a private practice, which, however great, is still inferior to his merits, enable its possessor to accumulate his experience, arrange its treasures, and leave to us as a legacy some, at least, of the knowledge he has hived, as well as the honourable name of which his professional and lineal descendants may be proud.

---

THE RESPIRATOR.

It is unnecessary for us to speak in favour of this ingenious and invaluable instrument. The experience of the profession has established its eminent utility as a preventive, if not also as a curative means. But we are anxious to introduce to our readers some modifications which its able inventor, Mr. Jeffreys, has made in it. *First,* the large sale, and improvements in the manufacture have enabled him to construct a cheap, yet most efficient instrument for the poor. The mechanic or the labourer may now obtain one for the low sum of seven shillings; while for females the price is only six shillings. This is an inestimable boon to the less affluent classes. It is unnecessary to point out the benefits that must accrue to many a family, from the head of it being enabled to pursue the occupation on which his and their subsistence depends. *Secondly,* a less important, yet not contemptible alteration has been made in the construction of the more expensive instruments. A light kind of scarf has been attached to the front, by which the *bizarrerie* of their appearance is removed. Our fair patients, at all events, will bless Mr. Jeffreys for this. In conclusion, we would entreat our professional friends to avail themselves of this admirable adjuvant to their previous stock of remedies.

